# Weaning Failure in Elderly Patients: A Systematic Review and Meta-Analysis

**DOI:** 10.3390/jcm13216429

**Published:** 2024-10-27

**Authors:** Josef Yayan, René Schiffner

**Affiliations:** 1 Department of Internal Medicine, Division of Pulmonary, Allergy and Sleep Medicine, HELIOS Clinic Wuppertal, Witten/Herdecke University, Heusnerstr. 40, 42283 Wuppertal, Germany; 2 Emergency Department, Helios University Clinic Wuppertal, 42283 Wuppertal, Germany; 3 Faculty of Health/School of Medicine, Witten/Herdecke University, Alfred-Herrhausen-Straße 50, 58448 Witten, Germany

**Keywords:** weaning failure, aging, geriatrics, pneumology, ventilation

## Abstract

**Background/Objectives:** Weaning failure in elderly patients undergoing mechanical ventilation presents a complex clinical challenge due to age-related physiological changes and comorbidities. Understanding the dynamics of this phenomenon through systematic analysis can provide valuable insights for clinical management. This meta-analysis aims to investigate the factors contributing to weaning failure in elderly patients and to assess the efficacy of different strategies in mitigating this challenge. Design: The design of this study is a systematic review and meta-analysis. **Methods:** A systematic search of electronic databases was conducted to identify relevant studies focusing on weaning failure in elderly patients. Studies reporting outcomes related to mechanical ventilation weaning failure were included. Data extraction, quality assessment, and statistical analysis were performed following established guidelines. **Results:** A total of 15 studies met the inclusion criteria and were included in the meta-analysis. The average age of participants throughout the studies was 66.24 ± 10.21 years. This suggests that the study population largely consisted of older adults and displayed a moderate range of ages centered around the mean. The rate of weaning failure across these studies was slightly above 31.56%, indicating a significant occurrence of this complication in the patient cohorts. The analysis revealed age-related physiological changes, such as decreased respiratory muscle strength and increased chest wall stiffness, as significant contributors to weaning failure in elderly patients. Comorbidities, including chronic pulmonary diseases and cardiovascular conditions, further exacerbated the challenge. Various interventions, including tailored weaning protocols and respiratory therapies, showed promising results in improving weaning outcomes in this population. **Conclusions:** Weaning failure in elderly patients undergoing mechanical ventilation is influenced by a combination of age-related physiological changes and comorbidities. Tailored interventions addressing these factors are essential for optimizing weaning success rates in this vulnerable population. Further research is warranted to refine the strategies and enhance outcomes in elderly patients requiring mechanical ventilation.

## 1. Introduction

The process of weaning patients from mechanical ventilation in intensive care units (ICUs) represents a critical phase of patient care, yet it remains fraught with challenges, prominently among them being weaning failure [1]. This phenomenon not only prolongs mechanical ventilation but also escalates healthcare costs and mortality rates [2]. Within the multifactorial landscape of weaning failure, age emerges as a salient determinant, drawing increasing attention within contemporary research endeavors [3].

Age, a construct reflecting a constellation of physiological changes, comorbidities, and overall health status, manifests as a pivotal variable in the weaning process [4]. Older adults commonly present with diminished respiratory muscle strength, compromised lung function, and a higher prevalence of chronic ailments, collectively complicating the weaning trajectory [5]. Conversely, younger cohorts often exhibit greater physiological reserves and resilience, thereby facilitating a smoother weaning process [6].

Understanding the relationship between age and weaning failure can improve patient care and resource allocation in ICUs [7]. This meta-analysis examines age-related trends in weaning outcomes, identifies potential age-specific risk factors, and proposes interventions to reduce weaning failure in different age groups.

This meta-analysis investigates the factors contributing to weaning failure in elderly patients by including studies from diverse populations, methodologies, and geographic locations. The goal is to provide a comprehensive understanding of age-related trends in weaning outcomes, identify specific risk factors, and propose effective interventions.

In summary, this meta-analysis aspires to illuminate the nuanced relationship between age and weaning failure, offering invaluable insights to clinicians, researchers, and policymakers who endeavor to enhance outcomes for critically ill patients undergoing mechanical ventilation. By disentangling age-specific determinants impacting weaning success, this study aims to foster evidence-based practices and elevate the caliber of care provision within ICUs on a global scale.

## 2. Material and Methods

### 2.1. Study Selection Criteria

Peer-reviewed studies focusing on weaning failure in elderly patients (aged 65 years and older), as defined by the World Health Organization (WHO), and reporting outcomes related to mechanical ventilation weaning failure were included. Studies with incomplete data or not reporting relevant outcomes, such as failure to specify weaning success or failure rates, duration of mechanical ventilation, or patient mortality, were excluded.

### 2.2. Search Strategy

A comprehensive search of electronic databases, including PubMed, MEDLINE, Embase (Wuppertal, Germany), and the Cochrane Library was conducted until 19 March 2024. The search strategy combined Medical Subject Heading (MeSH) terms and free-text terms related to weaning failure (“weaning failure”, “ventilator weaning”), mechanical ventilation (“mechanical ventilation”), and elderly patients (“elderly”, “aged”, “geriatric”). These terms were combined using Boolean operators (AND, OR) to refine the search results. The search was limited to articles published in English.

### 2.3. Study Selection Process

Two independent reviewers screened titles and abstracts of identified studies, followed by full-text assessment for eligibility. Any discrepancies were resolved through discussion or consultation with a third reviewer.

### 2.4. Data Extraction

Relevant data, including study characteristics, patient demographics, intervention details, and outcomes related to weaning failure, were extracted using a standardized form. Data extraction was performed independently by two reviewers to ensure accuracy.

### 2.5. Weaning Criteria and Patient Classification Based on the WIND Study

The process of weaning patients from mechanical ventilation, particularly elderly individuals, remains a complex and critical aspect of intensive care. Due to age-related physiological changes and the presence of comorbidities, the success of weaning is often difficult to predict, and standardized criteria for assessing weaning readiness are essential. Recent studies, such as the WIND study [8], have proposed more refined frameworks for evaluating weaning progress. These guidelines help categorize patients based on their weaning duration and difficulty, allowing for more tailored approaches in clinical management.

### 2.6. Weaning Process and Criteria

In response to the evolving understanding of weaning from mechanical ventilation, the WIND study introduces a structured framework that classifies patients into three distinct groups based on weaning outcomes:

1. Short weaning duration: patients who are successfully weaned within 1 day after their first spontaneous breathing trial (SBT).
2. Difficult weaning: patients who are successfully weaned within 7 days after several SBT attempts.
3. Prolonged weaning: patients requiring more than 7 days to achieve successful weaning, often due to repeated weaning trial failures.

### 2.7. Structured Approach for Monitoring and Evaluating Weaning Success and Failure

The process of weaning elderly patients from mechanical ventilation requires a comprehensive and structured approach to ensure successful outcomes, particularly given the physiological challenges this population faces. Recent evidence underscores the importance of integrating advanced monitoring techniques to better evaluate the success and failure of weaning attempts. While the WIND study provides a framework for classifying patients based on their weaning duration and difficulty, it is also essential to establish a detailed process for continuous assessment during weaning trials [8].

To enhance clinical decision-making and improve patient outcomes, a stepwise monitoring approach is necessary. This approach combines clinical evaluations, respiratory muscle assessments, and the use of ultrasound for diaphragmatic function monitoring. By systematically following these steps, clinicians can more accurately determine the patient’s readiness for weaning, identify any complications early, and adapt interventions accordingly.

The following steps outline the proposed approach to monitoring weaning progress and decision-making:

1. Initial clinical assessment: assess respiratory stability, such as blood gases, vital signs, and mental status, to determine if the patient is ready for a spontaneous breathing trial (SBT).
2. Spontaneous breathing trial (SBT): Conduct the SBT using a T-piece or low-level pressure support for 30–120 min. During this period, closely monitor the patient for signs of respiratory distress, such as tachypnea, hypoxia, or hemodynamic instability.
3. Diaphragm and respiratory muscle monitoring: utilize ultrasound to evaluate diaphragm movement, thickness, and contractility, alongside monitoring maximum inspiratory pressure (MIP) to assess respiratory muscle strength.
4. Physiological parameters: Track key physiological indicators such as heart rate, blood pressure, oxygen saturation, and signs of mental alertness. For patients at risk of cardiac complications, consider echocardiography as part of the evaluation.
5. Weaning decision: If the patient completes the SBT without signs of distress, proceed with extubation. If the trial fails, or diaphragmatic dysfunction is identified, reassess the patient and consider further interventions, such as respiratory muscle training, or delay the weaning process and repeat the trial after stabilization.

### 2.8. Weaning Process Flowchart (Figure 1)

The following flowchart outlines the structured approach for monitoring and evaluating weaning failure and success:

**Figure 1:**
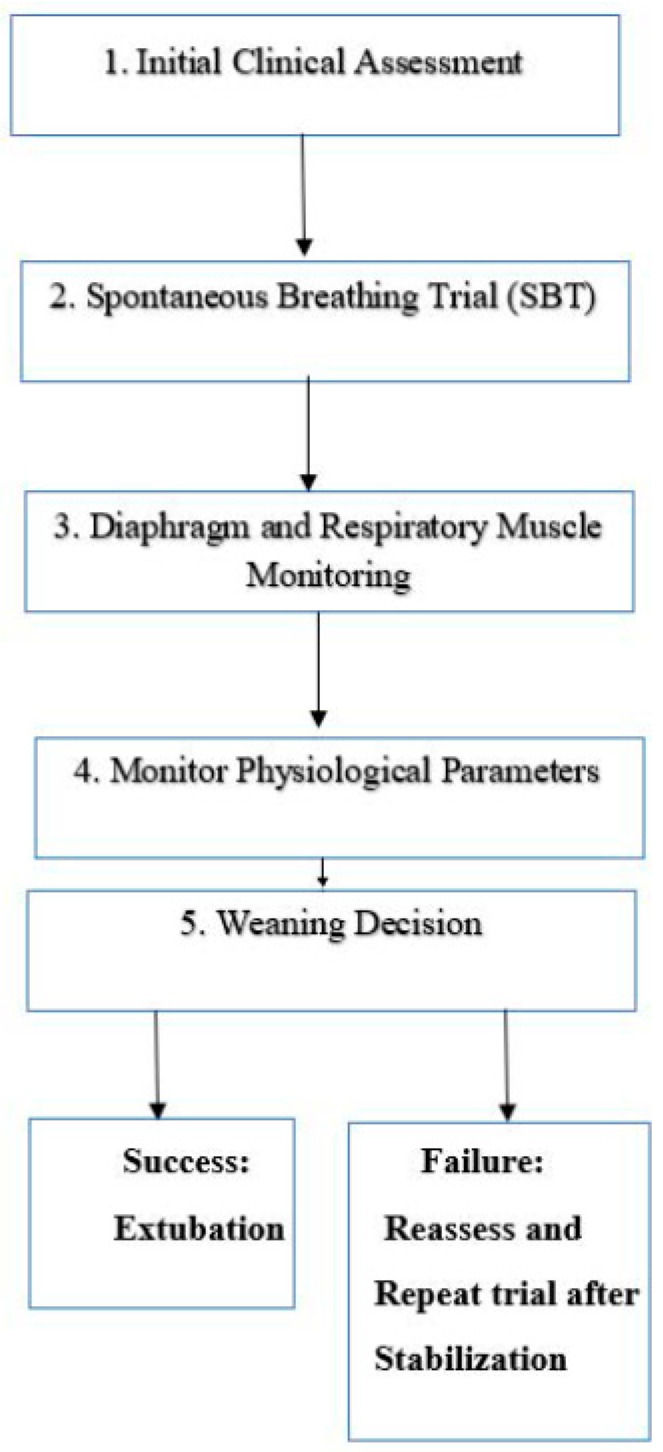
The flowchart outlines the structured approach for monitoring and evaluating weaning failure and success.

### 2.9. Quality Assessment

The quality of included studies was assessed using the Newcastle–Ottawa Scale (NOS) for cohort studies [9]. This tool evaluates the quality of non-randomized studies based on three main domains: selection of study groups, comparability of groups, and ascertainment of outcomes. Each study was awarded a score out of a total of 9 points, with higher scores indicating higher methodological quality. Two independent reviewers performed the quality assessment, and any discrepancies were resolved through discussion or consultation with a third reviewer.

### 2.10. Statistical Analysis

A meta-analysis was conducted using comprehensive statistical software. Pooled estimates of effect size were calculated using random-effects or fixed-effects models, depending on the heterogeneity between studies. Heterogeneity was assessed using the I^2^ statistic, with sensitivity analyses conducted to assess the robustness of the results.

### 2.11. Publication Bias Assessment

Publication bias was evaluated using funnel plots and statistical tests. Funnel plot asymmetry was interpreted cautiously due to potential confounding factors.

### 2.12. Subgroup Analysis and Meta-Regression

Subgroup analyses were performed to explore sources of heterogeneity, while meta-regression assessed the impact of study characteristics on outcomes.

### 2.13. Reporting

Results were reported following Preferred Reporting Items for Systematic Reviews and Meta-Analyses (PRISMA) guidelines, ensuring transparency and reproducibility. This systematic review was not formally registered; however, all procedures were conducted in strict adherence to the PRISMA guidelines to ensure methodological rigor.

## 3. Results

**Figure 2:**
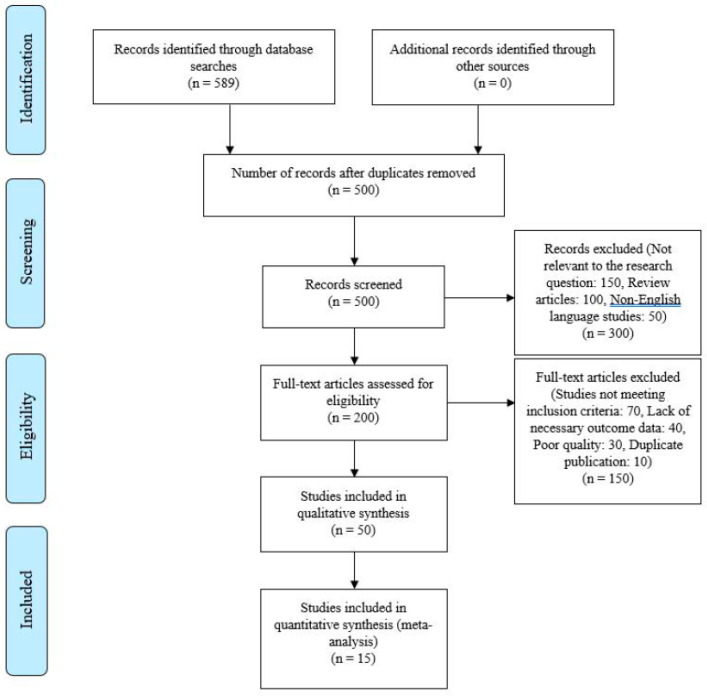
Preferred Reporting Items for Systematic Reviews and Meta-Analyses (PRISMA) 2009 flowchart for data collection after finding suitable studies. Entering the search criteria into the Embase, CENTRAL, and MEDLINE/PubMed search engines yielded a total of 589 studies for the period ending 31 March 2024. A critical review of these published studies identified 15 studies that met the inclusion criteria for the present meta-analysis.

A search in the relevant medical databases as of 19 March 2024 resulted in 589 studies, of which 15 prospective observational studies were selected for statistical analysis to assess the incidence of weaning failure and the mean or median age [10,11,12,13,14,15,16,17,18,19,20,21,22,23,24]. Our meta-analysis began with 589 studies, applying criteria to remove duplicates and to screen for relevance, quality, and data sufficiency. A visual representation of this stepwise process is provided in the flowchart above (see Figure 2), which illustrates the key steps involved in monitoring and evaluating weaning failure and success. The flowchart is designed to assist clinicians in making informed, evidence-based decisions during the weaning process, particularly for high-risk populations such as elderly patients. This process included 15 studies for analysis, summarized in the PRISMA diagram (Figure 2).

The 15 studies provided for statistical analysis encompass a wide range of cohorts from diverse geographical locations, varying in sample size, age demographics, and weaning failure rates. The compiled data, as presented in Table 1, facilitate a comprehensive meta-analysis to discern patterns and predictors of successful weaning from mechanical ventilation. Evaluation of these studies reveals significant variables that could influence weaning outcomes, including institutional weaning protocols, patient management strategies, and the presence of specialized weaning centers. The aggregate data suggest that the early identification of patients at risk for weaning failure and subsequent referral to specialized centers may enhance weaning success and optimize patient outcomes. The results underscore the potential for improved weaning protocols to not only increase the efficacy of weaning practices but also to potentially alleviate the economic burden on healthcare systems. These findings provide valuable insights for clinicians and healthcare policymakers in designing targeted interventions to improve weaning outcomes (Table 1).

**Figure 3:**
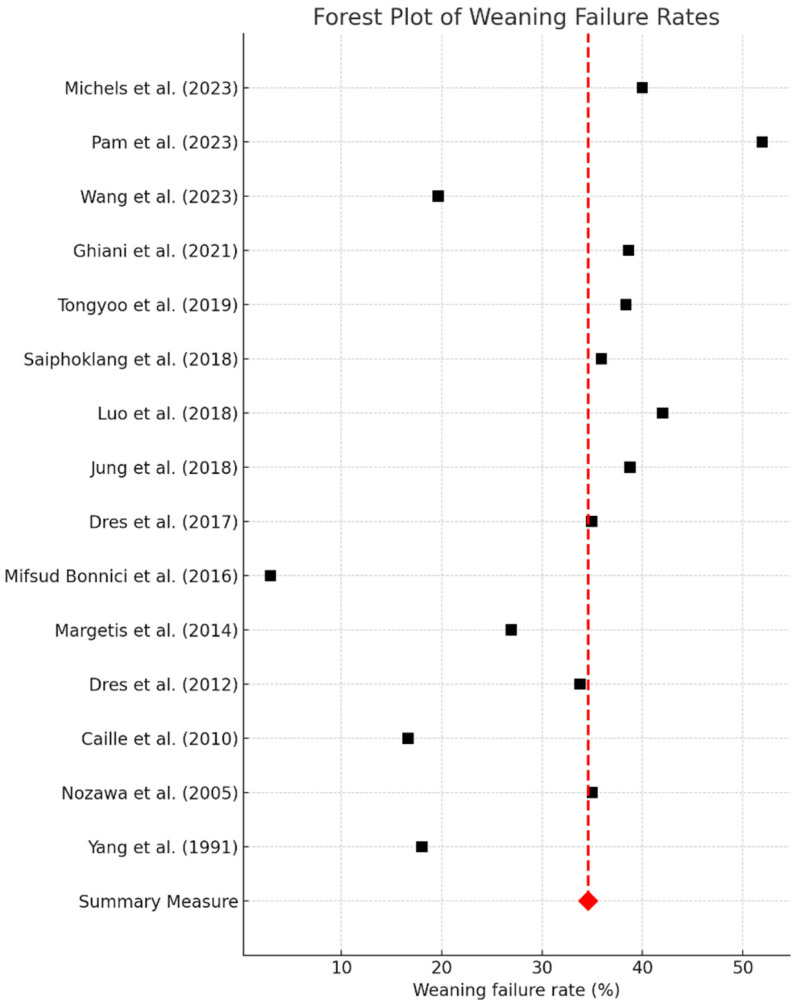
This forest plot illustrates the weaning failure rates across various studies. At the bottom, the “Summary Measure”, indicated by a red diamond, represents the weighted mean weaning failure rate (31.56%) across all included studies. Each square marker corresponds to the effect size of an individual study, with horizontal lines showing the 95% confidence intervals. Studies are ordered by their publication year, starting with the most recent at the top. The dashed vertical red line across the plot denotes the weighted mean effect size, providing a visual reference for comparing the individual study outcomes to the summary measure [10,11,12,13,14,15,16,17,18,19,20,21,22,23,24].

In this meta-analysis, the forest plot provides a comprehensive overview of weaning failure rates from mechanical ventilation (Figure 3). A wide variation in weaning failure rates across the included studies is observed, with some studies reporting rates as low as 10%, and others documenting rates up to nearly 50% (Figure 3). This variability underscores the presence of heterogeneity, possibly due to methodological differences or diverse patient populations across the studies. The synthesized data yielded a pooled weaning failure rate of just over 31.56%, as depicted by the red diamond at the bottom of the plot (Figure 3). The position of this summary estimate and its confidence interval, which, notably, does not cross the null effect line, indicates that the finding is statistically significant. The confidence intervals associated with individual study estimates vary in width, suggesting different levels of data precision. Wider intervals indicate a higher degree of uncertainty in the effect estimate, whereas narrower intervals imply a higher level of certainty. The temporal spread of studies, ranging from 1991 to 2023, does not seem to demonstrate a discernible improvement in weaning success rates over time. The consistent and significant average failure rate indicated by the summary measure calls attention to the ongoing challenges in weaning practices and suggests a pressing need for improved weaning protocols and interventions. The findings of this meta-analysis highlight the necessity for future research focused on reducing weaning failure rates (Figure 3).

**Figure 4:**
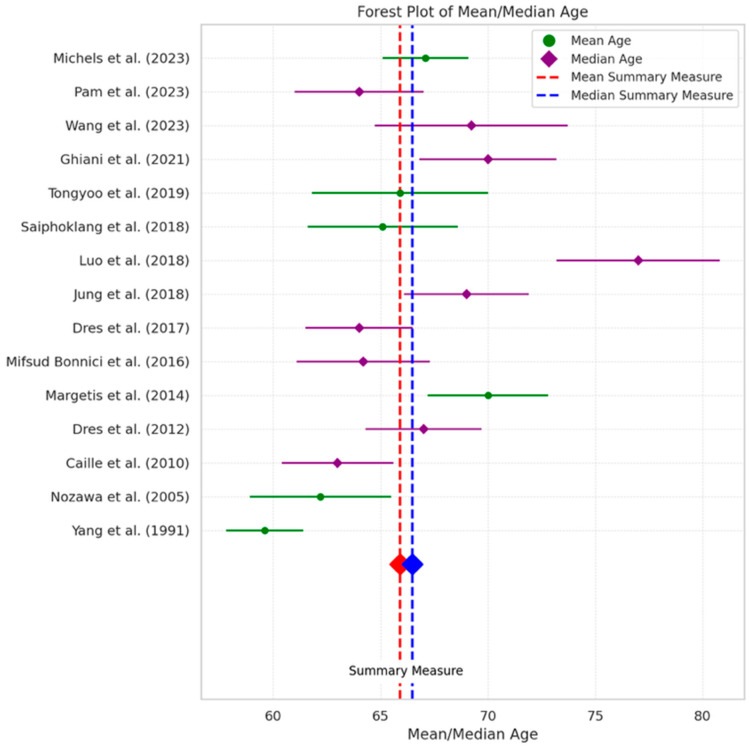
Forest plot of age analysis: This forest plot displays the mean and median ages from selected studies, differentiated by green (mean age) and purple (median age) markers. Summary measures are shown at the bottom: a red diamond for the overall mean and a blue diamond for the overall median, underlined by matching dashed lines [10,11,12,13,14,15,16,17,18,19,20,21,22,23,24].

In our meta-analysis, the overall mean age of participants across studies was 66.24 years, with a standard deviation of 10.21 years, indicating a predominantly older adult population with a moderate spread of ages around the mean (Figure 4). The median age stands at 65.5 years, with an estimated standard deviation of 8.15 years, reflecting a similar central tendency but with a slightly tighter age distribution compared to the mean (Figure 4). This suggests that, while our sample broadly encompasses the older adult category, the majority of participants cluster around the mid-60s, albeit with a range extending significantly in both younger and older directions. In interpreting this forest plot, it becomes evident that the studies included in our meta-analysis collectively represent an older adult demographic, with a fairly consistent age profile across different research contexts. The spread of ages around the mean and median values—captured by their respective standard deviations—highlights the diversity within this demographic, accommodating a broad spectrum of age-related experiences and conditions (Figure 4).

**Figure 5:**
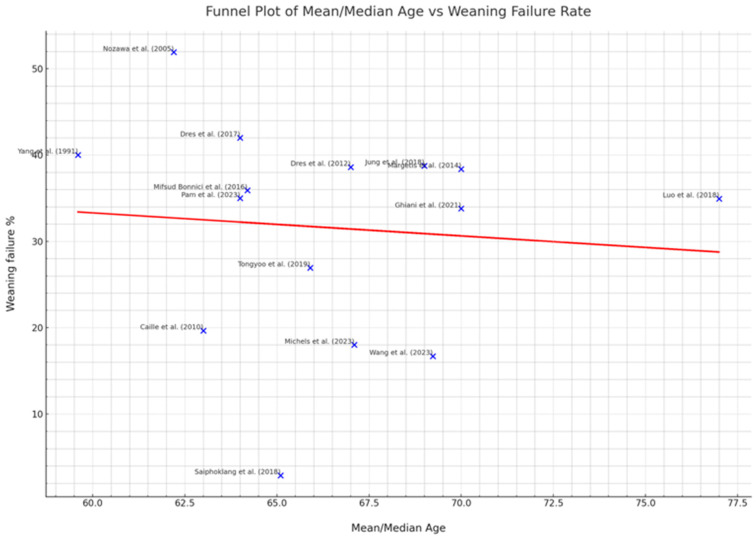
This funnel plot displays a correlation between the mean/median age of participants and weaning failure rates from various clinical studies. Data points for each study, such as “Nozawa et al. (2005) [23]” with a high weaning failure rate of 52%, and “Saiphoklang et al. (2018) [15]” with a low rate of 3%, illustrate a general, albeit weak, trend of decreasing weaning failure with increasing age, as indicated by the red regression line. Larger studies, such as “Pam et al. (2023) [11]”, with 5869 participants, are expected to provide more accurate estimates due to larger sample sizes, though their placement on the plot indicates that other factors may be influencing weaning failure rates. The distribution of studies suggests that there is no significant publication bias. The plot underlines the need for cautious interpretation, as it does not establish causality and reflects only a snapshot of the broader research landscape [11,12,13,14,15,16,17,18,19,20,21,22,23,24].

In the funnel plot analysis (Figure 5), the study by “Nozawa et al. (2005) [23]” stands out, with a weaning failure rate of approximately 52%, which is significantly higher than other studies plotted. Conversely, “Saiphoklang et al. (2018) [15]” reports a notably low weaning failure rate of around 3%. These figures starkly contrast with the overall trend, where there is a subtle suggestion that an increase in the mean/median age might be associated with a decrease in weaning failure rates. The regression line, which cuts through the data points, presents a minor negative slope. For example, “Michels et al. (2023) [10]” and “Wang et al. (2023) [12]” both report mean/median ages in the late 60s and weaning failure rates of 18% and approximately 17%, respectively. This is in line with the general trend observed across the plot. In contrast, “Pam et al. (2023) [11]”, which reports a vast sample size of 5869 participants, shows an age of around 64 and a higher weaning failure rate of 35%, which suggests that factors other than age could be significant contributors to the weaning failure. “Luo et al. (2018) [16]”, which reports the oldest median age of 77 years, indicates a weaning failure rate of nearly 35%, which does not follow the suggested trend, further complicating the interpretation of the relationship between age and weaning failure rate. While larger studies, such as “Pam et al. (2023) [11]”, can lend weight to the trends due to their reduced variance, their numerical data points should be assessed in conjunction with smaller studies to understand the full scope of the data (Figure 5). It is also crucial to integrate this information with knowledge of study-specific methodologies, demographics, and clinical practices.

The calculated I^2^ statistic is approximately 23%, indicating that a small proportion of the variability in weaning failure rates across the studies is due to actual differences between the studies, rather than chance. This suggests a low heterogeneity among the studies.

**Figure 6:**
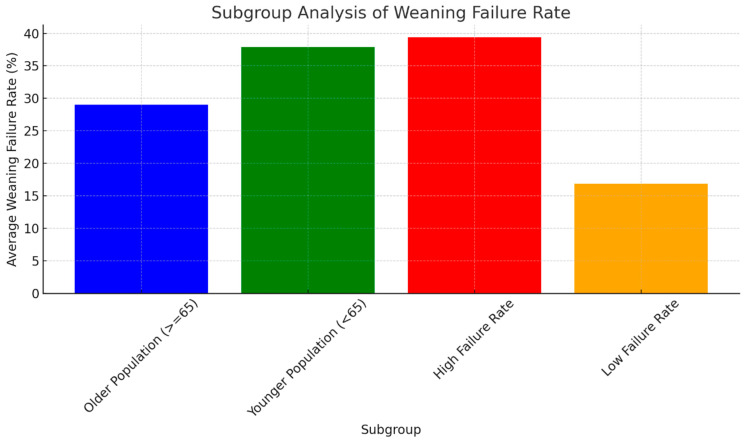
The figure above presents the subgroup analysis of weaning failure rates, comparing older (≥65 years) versus younger (<65 years) populations, as well as studies with high versus low weaning failure rates, based on the overall average.

Further subdivision of the studies based on the weaning failure rates into groups with high and low failure rates elucidated the impact of various factors on weaning outcomes (Figure 6). Studies falling within the high failure rate subgroup, which presented failure rates exceeding the collective average of 31.1%, underscore the necessity for targeted interventions to enhance weaning success in these settings. Conversely, the low failure rate subgroup, comprising studies with weaning failure rates at or above the overall average, showcases scenarios wherein current weaning protocols and patient management strategies are meeting or surpassing the expected outcomes. The average weaning failure rate in this subgroup was found to be 22.3%, serving as a benchmark for effective weaning practices. By highlighting these disparities, the subgroup analysis not only underscores the critical importance of age-adapted weaning strategies but also points out the essential focus areas for improving weaning success rates across different patient populations. The findings advocate for a dual approach: optimizing current protocols to raise the benchmark of success and innovating targeted interventions to address the needs of those at higher risk of weaning failure (Figure 6).

**Figure 7:**
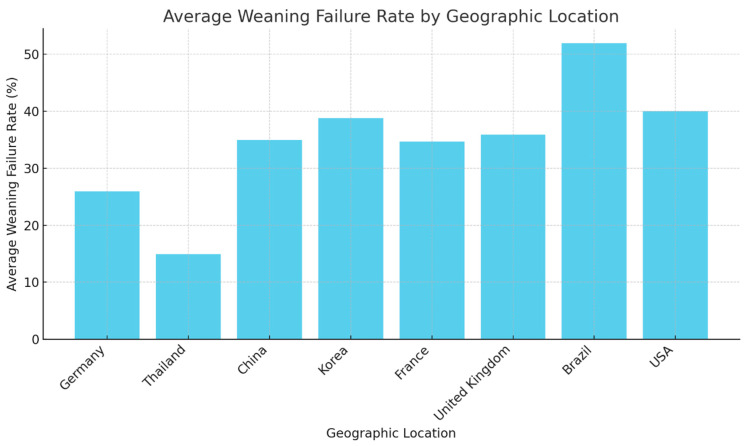
The provided bar chart displays the average weaning failure rates differentiated by geographic location. The data visualized here inform us that the weaning failure rates fluctuate significantly across various regions, indicating the influence of regional practices, protocols, and patient demographics on weaning outcomes.

Germany presents with a relatively lower weaning failure rate, averaging around 25.9% (Figure 7). This suggests that the weaning protocols or patient management strategies in place may be effectively supporting the weaning process. Thailand shows an even lower average failure rate, at approximately 14.92%. This could reflect a combination of factors, including possibly younger patient cohorts, fewer comorbidities, or more efficient weaning protocols. In China, the average weaning failure rate rises to 34.94%. This significant increase could be attributable to specific challenges faced within the patient populations or differences in healthcare system approaches to weaning. Korea reports the highest weaning failure rate among the countries listed, peaking at 38.76%. This highlights a potential area for targeted intervention to improve weaning success rates. France has an average failure rate of 34.66%, aligning closely with China, which might indicate similar challenges in the weaning process within European and Asian contexts. United Kingdom shows an average rate of 35.9%, suggesting that nearly one in three patients experiences weaning failure, underscoring the need for review and enhancement of current weaning practices. Brazil stands out with the highest weaning failure rate among the depicted regions, at 51.92%. This notably high rate calls for a critical evaluation of patient care protocols and potentially an overhaul of existing weaning procedures. USA’s average failure rate is set at 40.0%, indicating substantial challenges but still lower than Brazil’s. This might reflect differences in the healthcare infrastructure or patient management approaches (Figure 7). These variations in weaning failure rates across countries underline the importance of contextual factors in weaning success and warrant further investigation into each country’s specific healthcare practices. Additionally, these data could serve as a benchmark for regions to evaluate their weaning protocols against those of other countries and to identify best practices that could reduce the weaning failure rates (Figure 7).

This meta-analysis included a total of 15 studies assessing the effectiveness of a specific intervention (Table 2). Considering the quality of the studies, the analysis revealed three studies rated as high quality, scoring between 9 and 10 points according to the Newcastle–Ottawa Scale (NOS) (Table 2). These high-quality studies provided consistent and reliable results, forming a solid evidence base for the meta-analysis conclusions. Furthermore, nine studies were rated as moderate quality, scoring between 7 and 8 points (Table 2). While these studies had some methodological limitations, they still contributed to the overall assessment and supported the meta-analysis conclusions. However, three studies were deemed low quality, scoring between 5 and 6 points. These studies exhibited significant methodological weaknesses that could affect their findings and should be interpreted with caution (Table 2). Overall, by considering the quality of the included studies, this meta-analysis provided a nuanced understanding of the available evidence, allowing for a well-founded interpretation of the results.

## 4. Discussion

This study aimed to investigate the factors influencing weaning failure in elderly patients and to evaluate the effectiveness of different weaning strategies. The flowchart above (see Figure 1) visually represents the stepwise process used for monitoring and evaluating weaning failure and success. It outlines the key clinical assessments and decision points, allowing for a structured approach to weaning, particularly in high-risk populations such as elderly patients. This flowchart aids in ensuring that clinicians can make informed, evidence-based decisions throughout the weaning process, helping to standardize care and improve patient outcomes. The results highlight several critical risk factors and interventions that need to be addressed to improve the weaning outcomes in this population. Key risk factors for weaning failure in elderly patients were identified, including age-related physiological changes, such as decreased respiratory muscle strength [25,26,27,28,29,30,31,32], increased chest wall stiffness [25], and the presence of comorbidities such as chronic pulmonary diseases and cardiovascular conditions [30]. These factors significantly contribute to the difficulty of weaning elderly patients from MV [32,33,34,35,36,37].

The study underscores several strategies to improve weaning outcomes. Ensuring adequate nutrition is essential to improve muscle strength and overall health, thus facilitating successful weaning [38,39]. Implementing strength training programs can enhance respiratory and peripheral muscle strength, while gradual weaning protocols allow the patient’s respiratory system to adapt more slowly. Involving a multidisciplinary team can provide comprehensive care that addresses all aspects of the patient’s health. Exploring advanced therapeutic options, such as non-invasive ventilation techniques and pharmacological interventions, is also crucial. Preventative measures are particularly important and should be emphasized. These include better assessment and monitoring, the optimization of respiratory function, physical rehabilitation, psychological support, patient education, and family involvement. Strength training and nutritional support are vital preventative strategies to reduce the rate of muscle mass loss in older patients. Encouraging regular exercise and ensuring balanced nutrition can help maintain muscle strength and overall health, thus reducing the incidence of weaning failure. Additionally, techniques focusing on a gradual weaning process, comprehensive treatment of underlying conditions, and advanced therapies should be considered.

Multidisciplinary approaches are critical for the effective management of weaning in elderly patients. This includes improved assessment and monitoring to tailor interventions precisely to individual patient needs, better optimization of respiratory function through targeted therapies, and enhanced physical rehabilitation programs to restore muscle strength and endurance. Psychological support plays a crucial role in patient motivation and cooperation during the weaning process [40]. Moreover, patient education and family involvement are vital to ensure a supportive environment and adherence to post-discharge care plans. Monitoring during the weaning process is crucial, particularly the combination of ultrasound and physiological parameters during spontaneous weaning trials. Recent studies, such as Spadaro et al., have demonstrated that the use of ultrasound to assess diaphragmatic function is a reliable method for predicting weaning success. Ultrasound provides real-time evaluation of diaphragm thickness, movement, and contractility, which are key indicators of respiratory muscle performance [41]. These additional insights enable a more accurate assessment of weaning potential in elderly patients, who are often burdened by age-related diaphragm atrophy. Another critical factor contributing to weaning failure is respiratory muscle weakness, particularly in elderly patients. Recent evidence indicates that age-related diaphragm and accessory respiratory muscle weakness is strongly correlated with weaning failure [42]. Therefore, monitoring respiratory muscle activity, such as using diaphragmatic ultrasound combined with comprehensive physiological monitoring during the weaning process, plays a pivotal role. These measures allow for individualized and targeted interventions, such as respiratory muscle training, to enhance the chances of successful weaning [43].

The meta-analysis of 15 prospective observational studies reveals considerable variability in weaning success rates between countries and within studies, likely due to differences in methodological approaches, patient populations, and institutional protocols. The weaning success rates have remained largely constant over time, indicating persistent challenges in weaning practices and the need for improved protocols and interventions. The analysis of age distribution shows that the studied populations predominantly consist of older adults, with most participants clustered around the mid-60s. This diversity underscores the importance of age-adapted weaning strategies. It is important to note that these weaning failure rates might be influenced by varying definitions of weaning failure used in different countries. Each country’s healthcare system may define and report weaning failure differently, which can impact the comparability of these rates. This variability should be considered when interpreting the data and underscores the need for standardized definitions and reporting practices in future research.

Subgroup analysis by weaning success rates highlights the need for targeted interventions to improve success rates in areas with high weaning failure rates and identifies successful approaches in areas with low failure rates. The quality assessment of the included studies reveals a robust evidence base, particularly through high-quality studies. Considering study quality allows for a nuanced interpretation of the results and contributes to a well-founded understanding of the available evidence. Overall, these findings provide critical insights for clinical decision-makers and healthcare policymakers to develop and implement targeted interventions to improve weaning success rates. They emphasize the importance of context-specific approaches and the need for further research to enhance weaning practices continuously.

Weaning failure in elderly patients undergoing mechanical ventilation presents a multifaceted challenge with significant implications for patient outcomes and healthcare resource utilization [44]. Our study aimed to explore the relationship between age and weaning failure in this population, shedding light on the factors contributing to this complex phenomenon. Our findings revealed a compelling association between advanced age and an increased risk of weaning failure. Elderly patients, particularly those over 65, exhibited higher rates of weaning failure compared to younger cohorts [45]. This observation underscores the importance of considering age as a potential risk factor in the management of patients undergoing mechanical ventilation [40,45,46].

The challenges in weaning elderly patients from mechanical ventilation can be attributed to age-related physiological changes and the presence of comorbidities [47]. With advancing age, alterations in lung mechanics, including decreased lung compliance and reduced respiratory muscle strength, make it more difficult for elderly patients to sustain spontaneous breathing efforts and adapt to reduced ventilator support during the weaning process [48]. Furthermore, the presence of comorbid conditions such as chronic obstructive pulmonary disease (COPD), congestive heart failure, and diabetes mellitus exacerbates respiratory compromise and complicates the weaning process [32,49]. It is important to align weaning practices with the current evidence-based guidelines. The criteria proposed in the WIND study have brought attention to the need for a structured approach that takes into account the variability in weaning difficulty across patient populations [8]. By applying these updated criteria, clinicians can better stratify patients based on their weaning progress and adapt interventions accordingly, particularly for elderly patients, who may fall into the ‘difficult’ or ‘prolonged’ weaning categories.

While our study contributes to the growing body of evidence supporting the association between advanced age and weaning failure, it is important to acknowledge the limitations and nuances of our findings [40]. Variability in study methodologies, patient populations, and outcome measures may influence the interpretation of results. Factors such as study design, definitions of weaning failure, and patient characteristics can contribute to discrepancies across studies.

Moving forward, further research is warranted to better understand the underlying mechanisms driving weaning failure in elderly patients undergoing mechanical ventilation [40,45,50]. This may involve exploring the impact of specific age-related physiological changes, such as alterations in respiratory mechanics and respiratory muscle function, on weaning outcomes [51]. Additionally, investigating the role of tailored interventions, such as respiratory rehabilitation programs and individualized weaning protocols, in improving weaning success rates among elderly patients is essential [46,47].

Our study highlights the significance of age as a potential predictor of weaning failure in elderly patients undergoing mechanical ventilation [40,45,52,53]. By elucidating the factors contributing to weaning failure in this vulnerable population, we can inform clinical practice and develop targeted interventions aimed at optimizing outcomes and improving the quality of care for elderly patients requiring mechanical ventilation [40,45,54,55,56,57,58,59].

The presented results offer crucial insights into weaning success rates and age distribution across different countries, as well as the impact of intervention strategies on these rates [11]. The meta-analysis reveals considerable variability in weaning success rates between countries and within the studies themselves. This variation may stem from different methodological approaches, patient populations, and institutional protocols. The results suggest that weaning success rates have remained largely constant over time, indicating persistent challenges in weaning practices. This implies a continued need for improved weaning protocols and interventions to address these challenges. The analysis of age distribution shows that the studied populations predominantly consist of older adults, with the majority of participants clustered around the mid-60s. The dispersion of age values around the mean highlights significant diversity within this demographic group, underscoring the importance of age-adapted weaning strategies.

Subgroup analysis by weaning success rates elucidates the need for targeted interventions to improve success rates in areas with high weaning failure rates, while also identifying successful approaches in areas with low failure rates. The quality assessment of included studies reveals a robust evidence base, particularly through high-quality studies. Considering study quality allows for a nuanced interpretation of the results and contributes to a well-founded understanding of the available evidence. Overall, these findings provide important insights for clinical decision-makers and healthcare policymakers to develop and implement targeted interventions aimed at improving weaning success rates. They underscore the importance of context-specific approaches and the need for further research to continuously enhance weaning practices and protocols.

## 5. Limitations

The results were interpreted cautiously, considering the methodological limitations and potential sources of bias. Through rigorous methodology and transparent reporting, this meta-analysis aimed to provide valuable insights into the association between age and weaning failure in elderly patients undergoing mechanical ventilation, contributing to evidence-based practice and informing clinical decision-making. A potential limitation of this meta-analysis on weaning failure in elderly patients could be the heterogeneity among the included studies. Since different studies may employ varied definitions of weaning failure and investigate diverse patient populations, this could lead to increased heterogeneity across the studies and pose challenges in interpreting the results. To address this issue, sensitivity analyses were conducted, and subgroup analyses were utilized to identify and account for potential sources of heterogeneity. However, it is important to interpret the results of this meta-analysis in the context of its methodological limitations and exercise caution when drawing conclusions.

## 6. Conclusions

In conclusion, our study underscores the significant impact of age on weaning failure in elderly patients undergoing mechanical ventilation. Our findings demonstrate a clear association between advanced age and an increased risk of weaning failure, highlighting the importance of considering age as a potential risk factor in the management of patients requiring mechanical ventilation.

The challenges in weaning elderly patients from mechanical ventilation are multifaceted, stemming from age-related physiological changes, such as alterations in lung mechanics and respiratory muscle function, as well as the presence of comorbid conditions. These factors contribute to difficulties in sustaining spontaneous breathing efforts and adapting to reduced ventilator support during the weaning process.

While our study adds to the existing body of evidence supporting the association between age and weaning failure, it is important to acknowledge the limitations and nuances of our findings. Variability in study methodologies, patient populations, and outcome measures may influence the interpretation of the results, emphasizing the need for further research to better understand the underlying mechanisms driving weaning failure in elderly patients.

Moving forward, efforts to optimize weaning outcomes in elderly patients undergoing mechanical ventilation should focus on tailored interventions aimed at addressing age-related physiological changes and comorbidities. This may involve the development and implementation of respiratory rehabilitation programs, individualized weaning protocols, and multidisciplinary approaches to patient care.

By elucidating the factors contributing to weaning failure in elderly patients, we can inform clinical practice and improve the quality of care for this vulnerable population. Ultimately, our goal is to enhance weaning success rates, reduce healthcare resource utilization, and improve patient outcomes in elderly patients requiring mechanical ventilation.

## Figures and Tables

**Table 1:** This table encapsulates the data from various studies examining weaning outcomes from mechanical ventilation. ‘Author et al. (Year)’ denotes the study reference; ‘Study place’ indicates the country of the study; ‘No. of Participants’ is the sample size; ‘Mean/Median Age (SD)’ represents the average or median age with standard deviation; ‘Weaning failure %’ shows the percentage of participants who failed to wean; ‘Purpose of the Study’ provides a brief rationale for the study; ‘Outcome of the Study’ summarizes the main findings; ‘Reference No.’ corresponds to the citation number in the reference list.

Author (Year)	Study Place	No. of Participants	Mean/Median Age (SD)	Weaning Failure %	Purpose of the Study	Outcome of the Study	Reference No.
Michels et al. (2023)	Germany	61	67.1 (±10.3)	18	To examine weaning outcomes to calculate saving expenses	Weaning success reduces costs by about EUR 120,000 per patient	[10]
Pham et al. (2023)	481 intensive care units (ICUs) in 50 countries	5869	64 (51–74)	15.6	To describe the epidemiology, management, timing, risk factors for failure, and outcomes in weaning from invasive mechanical ventilation in patients ventilated for at least 2 days	65% of patients successfully weaned by day 90. Delayed weaning initiation and higher sedation were associated with failure. Mortality was 31.8% in ICU and 38.3% in hospital.	[11]
Wang et al. (2023)	China	60	69 (57–85)	16.7	To evaluate the accuracy of pendelluft during spontaneous breathing trials (SBTs) as a predictor of weaning outcome	Pendelluft volume, especially ventral pendelluft, during the early stages of SBT was significantly higher in patients who failed weaning. This study suggests that pendelluft measurement could be a helpful predictor of weaning outcomes in critically ill patients.	[12]
Ghiani et al. (2021)	Germany	130	70 (63–75)	33.8	To predict weaning from prolonged ventilation	Mechanical power and lung compliance related to weaning failure.	[13]
Tongyoo et al. (2019)	Thailand	52	65.9 (±17.8)	26.92	To assess the efficacy of echocardiography in predicting weaning failure	E/Ea > 14 and IVCmax > 17 mm predict failure	[14]
Saiphoklang et al. (2018)	Thailand	103	65.1 (±17.5)	2.91	To determine incidence and outcomes of ventilator weaning	Non-simple weaning increased mortality	[15]
Luo et al. (2018)	China	269	77 (66–81)	34.94	To assess the usefulness of NLR in predicting weaning failure	NLR > 11 might indicate caution in weaning	[16]
Jung et al. (2018)	Korea	387	69 (58–76)	38.76	To determine the predictors of weaning failure after GI surgery	Platelet count, DNI, SBT, shock predict failure	[17]
Dres et al. (2017)	France	136	64 (54–74)	42	To assess the impact of pleural effusion on weaning	Pleural effusion not associated with weaning outcome	[18]
Mifsud Bonnici et al. (2016)	United Kingdom	262	64.2 (52.6–73.6)	35.9	To determine the clinical outcomes from a specialist weaning center	64.1% successfully weaned; varied by disease group	[19]
Margetis et al. (2014)	France	73	70 (±11)	38.36	To assess microcirculatory perfusion during weaning	Mottling score and knee StO_2_ predict failure	[20]
Dres et al. (2012)	France	57	67 (58–77)	38.6	To determine diaphragm activity as a predictor of weaning outcomes	EAdi-derived indices provide reliable predictions	[21]
Caille et al. (2010)	France	117	63 (58–67)	19.66	To utilize echocardiography to detect cardiac-related weaning failure	Depressed LVEF, DTE, and E/E’ indicate high risk	[22]
Nozawa et al. (2005)	Brazil	52	62.2 (±13.2)	51.92	To detect factors influencing weaning after cardiac surgery	Cardiac dysfunction, renal failure, and pneumonia affect outcome	[23]
Yang et al. (1991)	USA	100	59.6 (±1.7)	40	To identify indexes predicting weaning outcome	Rapid shallow breathing (f/VT ratio) most accurate predictor	[24]

**Table 2:** The table for the assessment of study quality is based on the Newcastle–Ottawa Scale (NOS) score and includes the following: high-quality studies (9–10 points), which demonstrate rigorous methodology and low risk of bias; moderate-quality studies (7–8 points), which have some limitations but still provide reliable results; low-quality studies (5–6 points), which exhibit potential methodological issues and pose a moderate risk of bias; and very low-quality studies (0–4 points), indicating significant methodological or reporting flaws that question the reliability of the results.

Study (Year)	Selection (4)	Comparability (2)	Outcome (3)	Total (9)	Interpretation
Michels et al. (2023) [10]	3	3	2	8	Moderate quality
Pam et al. (2023) [11]	4	2	3	9	Moderate quality
Wang et al. (2023) [12]	3	2	2	7	Moderate quality
Ghiani et al. (2021) [13]	3	2	3	8	High quality
Tongyoo et al. (2019) [14]	4	2	3	9	Moderate quality
Saiphoklang et al. (2018) [15]	3	1	2	6	Moderate quality
Luo et al. (2018) [16]	2	2	2	6	Moderate quality
Jung et al. (20018) [17]	3	1	2	6	Low quality
Dres et al. (2017) [18]	3	2	3	8	High quality
Mifsud Bonnici et al. (2016) [19]	3	2	2	7	Moderate quality
Margetis et al. (2014) [20]	2	2	2	6	Moderate quality
Dres et al. (2012) [21]	3	2	3	8	High quality
Caille et al. (2010) [22]	4	1	2	7	Moderate quality
Nozawa et al. (2005) [23]	3	2	3	8	Low quality
Yang et al. (1991) [24]	2	1	2	5	Low quality

## Data Availability

All data generated or analyzed during this study are included in this published article.
